# Editorial: Advances and Challenges of Carrier Architectures for Bioactive Delivery Systems

**DOI:** 10.3389/fchem.2021.739946

**Published:** 2021-09-02

**Authors:** Ming Miao, Srinivas Janaswamy

**Affiliations:** ^1^ State Key Laboratory of Food Science and Technology, Jiangnan University, Wuxi, China; ^2^ Department of Dairy and Food Science, South Dakota State University, Brookings, SD, United States

**Keywords:** bioactive compounds, delivery systems, delivery vehicles, architecture, bioavailability, solubility

Bioactive compounds with unique functional attributes such as hypoglycemic, hypolipidemic, antidiabetic, anti-inflammatory, antimicrobial and antioxidant are of growing interest due to their widespread food, nutraceutical, medicine, cosmetics, chemical and agricultural applications. Their commercial use, however, is sparse due to poor solubility, instability and susceptibility to light, heat, oxygen, acid and alkaline conditions. Their low and inconsistent bioavailability and efficacy further hamper the large-scale utilization. To overcome these predicaments, design and development of innovative and high-performance protection and delivery systems are in great demand. Toward this end, food-grade materials gained much attention due to their intrinsic biocompatibility, biodegradability and reduced environmental impact (Chen et al., 2016; Miao and Hamaker, 2021). Nanostructural materials nanoemulsions, nanohydrogel, nanocomplexes, nanofibers and nanoparticles are also expanding the current understanding (Ye et al., 2018; McClements, 2020; Shi et al., 2020).

Despite significant progress and knowledge advancement, there are many lingering issues and challenges, for example, need for more diverse source of environmentally friendly materials to build delivery cargos, delivery systems design with multi-functionality, facile and low manufacture cost fabrication protocols along with cost-effective analytical characterization tools on bioactive bioavailability, to name a few. In this special issue, researchers around the world were invited to contribute their seminal works on the bioactive compounds delivery with an emphasis on the current updates and design strategies. Experimental data and reviews are reported along with nanotechnology-based delivery systems to administer the lipophilic ingredients with improved solubility and bioavailability.

A nano-formulation of zein and dextran sulfate sodium (DSS) binary complex for sustained delivery of quercetin has been reported by Wang et al. These nanoparticles possess smooth spherical shape with a size range of 180–250 nm. The DSS located on the hydrophilic exterior reduces the surface hydrophobicity and increases the encapsulation efficiency with improved bioavailability of quercetin, which certainly stands-out as an attractive carrier system for bioactive compounds. A bird’s eye view on various aspects of lipid nanoparticles (solid lipid nanoparticles and nanostructure lipid carriers) as carriers of bioactive molecules, including synthesis, characterization, advantages, disadvantages, toxicity and applications have been narrated by Dhiman et al. A comprehensive literature review on the recent advances of protein/peptide-based nanohydrogels toward bioactive delivery, including preparation, biophysiochemical aspects and applications in diverse disciplines like in drug delivery, immunotherapy, intracellular delivery, nutraceutical delivery, cell adhesion and wound dressing has been exemplified by Chander et al. Nanohydrogels are excellent biomaterials with three-dimensional cross-linked networks and are composed of diverse types of proteins with high biocompatibility and metabolic degradability. The advantages, limitations, overview of clinical potential, toxicity aspects, stability issues, and future perspectives of protein nanohydrogels have also been discussed. Acevedo-Fani et al. reviewed the nature-assembled structures for delivery of bioactive compounds and their potential in functional foods. They bring out the nature-origin structure and biological function of different nature-assembled carriers, namely casein micelles, milk fat globules and oleosomes along with preparation/isolation methods, advantages and challenges, and the behavior of these structures during digestion.

This collection is deemed to provide a central venue for researchers and scientists with interests to create high quality, safer, nutritious, sustainable and more abundant delivery systems supply of bioactive compounds.

## References

[B1] Chen J. Miao M. Campanella O. Jiang B. Jin Z. (2016). Biological Macromolecule Delivery System for Improving Functional Performance of Hydrophobic Nutraceuticals. Curr. Opin. Food Sci. 9, 56–61. 10.1016/j.cofs.2016.08.002

[B2] McClements D. J. (2020). Nano-enabled Personalized Nutrition: Developing Multicomponent-Bioactive Colloidal Delivery Systems. Adv. Colloid Interf. Sci. 282, 102211. 10.1016/j.cis.2020.102211 32721626

[B3] Miao M. Hamaker B. R. (2021). Food Matrix Effects for Modulating Starch Bioavailability. Annu. Rev. Food Sci. Technol. 12, 169–191. 10.1146/annurev-food-070620-013937 33395539

[B4] Shi Y. Ye F. Lu K. Hui Q. Miao M. (2020). Characterizations and Bioavailability of Dendrimer-like Glucan Nanoparticulate System Containing Resveratrol. J. Agric. Food Chem. 68, 6420–6429. 10.1021/acs.jafc.0c01315 32396340

[B5] Ye F. Miao M. Cui S. W. Jiang B. Jin Z. Li X. (2018). Characterisations of Oil-In-Water Pickering Emulsion Stabilized Hydrophobic Phytoglycogen Nanoparticles. Food Hydrocolloids 76, 78–87. 10.1016/j.foodhyd.2017.05.003

